# The interaction between gut microbiome and nutrients on development of human disease through epigenetic mechanisms

**DOI:** 10.5808/GI.2019.17.3.e24

**Published:** 2019-09-26

**Authors:** Ho-Sun Lee

**Affiliations:** Forensic Toxicology Division, Daegu Institute, National Forensic Service, Chilgok 39872, Korea

**Keywords:** disease development, fetal reprogramming, gut microbiome, nutrition

## Abstract

Early environmental exposure is recognized as a key factor for long-term health based on the Developmental Origins of Health and Disease hypothesis. It considers that early-life nutrition is now being recognized as a major contributor that may permanently program change of organ structure and function toward the development of diseases, in which epigenetic mechanisms are involved. Recent researches indicate early-life environmental factors modulate the microbiome development and the microbiome might be mediate diet-epigenetic interaction. This review aims to define which nutrients involve microbiome development during the critical window of susceptibility to disease, and how microbiome modulation regulates epigenetic changes and influences human health and future prevention strategies.

## Introduction

In 1986, David Barker proposed the Developmental Origins of Health and Disease (DOHaD) hypothesis, denoted fetal reprogramming, that the environmental stimuli such as nutrients during vulnerable development stages may permanently program change of organ structure and function of the offspring toward the development of non-communicable diseases [1]. Since Barker’s observation, denoted fetal programming, it is widely recognized that nutrient during pregnancy and lactation is one of the modifiable risk factors for adult chronic diseases including diabetes and cardiovascular diseases [2]. Following the DOHaD concept, early-life environmental factors such as nutrients may modulate the development of the gut microbiota [3]. It is postulated that the gut microbiome can be shaped by nutrients, which induce epigenetic changes prior to fetal and infant programming of disease.

In this review, we describe how early nutrients link microbiome modulation and influence the early epigenome. We refer the term “early-life” to the period from pregnancy to 2 years of age. Moreover, we assess the early-life gut microbiome as epigenetic modifiers are associated with early-onset or adult diseases including metabolic disorders. In detail, section 2 reviews the regulation of epigenetic mechanisms for metabolic signaling through nutrients and their metabolites as epigenetic substrates or cofactors. Section 3 provides several early environmental factors that may modulate early microbial communities. Section 4 describes the effects of each nutrient on gut microbiome changes in early-life through epigenetic mechanisms and the potential role of the gut microbiome in adult disease.

## The Link between Dietary Epigenetic Modulator and Metabolic Processes

Epigenetic changes by environmental conditions enhance cellular plasticity [4]. Genomic DNA is packed into chromatin with histone protein and the changes of chromatin structure participate in the cellular processes in response to physiological signals. Chromatin structure is determined by epigenetic modification resulting in transcription and replication types of machinery and therefore influences biological consequences [5]. Epigenetic changes produce DNA, histone and chromatin modifications including methylation, acetylation, phosphorylation, sumoylation, and ubiquitination with modifying enzymes (e.g., methyltransferases) [6]. Non-coding RNA is an epigenetic mark that mediates epigenetic modification by environmental factors such as diet. Modification of chromatin architecture by epigenetic marks can be heritable and modify the risks of disease in later life (Table 1) [7-17].

Several metabolites or cofactors of tricarboxylic acid (TCA) cycles involved in methylation or demethylation of histone [18]. The epigenetic modifying enzymes use some energy metabolites in one-carbon metabolism with S-adenosyl-methionine (SAM) and TCA cycles. SAM is generated through one-carbon metabolism and a key factor for epigenetic processes. DNA methylation requires the SAM as a methyl donor, while ten-eleven translocation and Jumonji C domain-containing proteins-mediated demethylation requires TCA cycle metabolite α-ketoglutarate and Fe^2+^ as a cofactor. DNA and histone demethylation have the potential to regulate diverse physiological functions, including metabolic signaling [19].

There are several known dietary methyl donors including folate, choline, and vitamin B12 participate in one-carbon metabolism and these nutrients have been examined for the epigenetic regulation in animal and human studies [2]. Methyl donors are related to energy production and amino acid metabolism, therefore nutritional imbalance as the disruption of the one-carbon cycle may participate in the development of metabolic diseases. Besides, many studies have shown that the mechanism of developmental reprogramming is involved in dietary availability of methyl donors [2].

Histone (de)methylation is one of the mechanisms involved in several diseases including metabolic syndrome and cancer [20]. Lysine methylation of a specific site of H3 acts both transcriptional activation (H3K4) and silencing (H3K9, H3K27) for regulating gene expression by a degree of methylation within the histone tail. The H3K9 specific demethylase JmjC domain-containing histone demethylase 2A (JHDM2A) was well known to an important regulator of fatty acid metabolism, therefore loss of function of JHDM2A resulted in obesity and hyperlipidemia [21,22]. H3K4 demethylase LSD1 using flavin adenine dinucleotide (FAD) as a cofactor, epigenetically represses energy expenditure, which facilitates energy storage as triglycerides in white adipocytes [23]. FAD is also related to the TCA cycle, oxidative phosphorylation, and fatty acid β-oxidation with riboflavin (vitamin B2) in the cytoplasm and mitochondria [24].

Arginine methylation of histone has been rapidly highlighted over the years for the role of diseases [9]. Protein arginine methyltransferases (PRMTs) with SAM as a methyl donor, transfer of methyl groups to specific arginine residues in histone and nonhistone protein substrates, resulting in the byproduct S-adenosyl-homocysteine (SAH). The SAM/SAH ratio can reflect the cellular methylation potential, which interacted with PRMTs. Peroxisome proliferator-activated receptor gamma coactivator 1α (PGC-1α; encoded by the PPARGC1A) serves as a transcriptional coactivator in mitochondria and has a function in oxidative metabolism. PRMT1 mediated cytosine methylation in PGC-1α which is expressed in brown adipose tissue involved in energy metabolism and diabetes [25].

Acetylation of histone H3 and H4 is mediated by acetyl-CoA and NAD+ for cellular metabolism. Lysine acetylation occurs in proteins involved in glycolysis, pyruvate metabolism and the TCA cycle [26]. Sirtuin 1 (SIRT1), a deacetylase depending on NAD+ has emerged as a key metabolic sensor linking to metabolic homeostasis, and SIRT1 participates the regulation of glucose homeostasis under nutrient deficiency [27]. For example, SIRT1 modulates acetylation on H3K9/K14 at circadian clock-controlled gene promoters through a high-fat diet [28]. It is reported that acetyl-CoA dependent histone acetylation may have an important role in the cellular assessment of metabolic states and circadian clock [29]. Therefore, nutrients (e.g., pyruvate, fatty acids, or branched-chain amino acids) involved in acetyl-Co A levels can modulate metabolic signaling.

Nutrients driven O-GlcNAc (N-acetylglucosamine) glycosylation has emerged as an important chromatin modification process [30,31]. O-GlcNAc transferase (OGT) is a key enzyme through protein glycosylation in the Hexosamone Biosynthetic Pathway. Several studies reported that nutrients-sensing GlcNAcylation of histone proteins has involved in chromatin remodeling and regulation of biological processes [32]. Chromatin GlcNAcylation depend on the cellular processes with glucose, glutamine, fatty acid and ATP metabolism. High fat diet induced insulin resistance, hyperphagia and obesity through O-GlcNAc cycling [33,34]; thus, OGT can be a sensor for adipose to brain axis to target obesity [34].

## Environmental Factors Involved in Early-Life Development of Microbiome

The early-life microbiota contains a unique microbial communities consisting of numerous bacteria and viruses. This microbiota has identified by using different kinds of technologies including 16S rRNA sequencing. The gut microbiota is linked to risk for various conditions in inflammatory diseases, asthma, obesity, glucose intolerance, and type 2 diabetes [35-37].

Over the last decades, the paradigm of a sterile condition *in utero* is shifting to the possibility of the prenatal maternal-fetal coexist with commensal and symbiotic microbes [38]. Recent studies also support a prenatal microbial milieu through bacterial presentation in placenta, amniotic fluid, umbilical cord, and meconium [39]. In addition, there are emerging reports of the prenatal microbial composition on fetal and postnatal development. However, concerns have raised by molecular based approaches, therefore it is needed for appropriate controls to account for DNA contamination or bacterial viability [40].

**Fig. 1.**
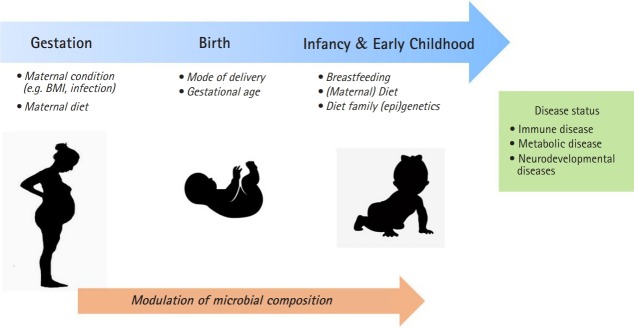
The critical window in early-life for microbiome modulation may influence on development of diseases later in life. BMI, body mass index.

A maternal condition during pregnancy and postnatal period can provide a critical window for susceptibility to microbiome development through the environmental factors such as mode of delivery and maternal diet (Fig. 1). The delivery mode has a crucial function in the early gut microbiota composition. Infants by vaginally delivery have higher levels of intestinal *Bacteroides, Lactobacilli*, and *Bifidobacterium*, which are commonly present in vaginal route, whereas infants by caesarean section (C-section) have higher level of *Enterococcus*, and *Clostridium* from skin, oral or hospital environment [41]. C-section born infants have shown to an increased risk of immune disorder such as asthma and allergy and obesity [42,43]. It has been revealed that neonates born by C-section exhibited significantly higher global DNA methylation levels in leucocytes [44] and CD34^+^ cell [45] compared with those born vaginally.

Gestational age is another important influencer for gut microbiome development [7,8]. It was reported that the gut microbiota of preterm infants has shown to delayed colonization by limited microbial diversity and ths risk of gut dysbiasis. The gut microbiota composition of preterm infants has *Enterobacter, Enterococcus, Escherichia*, and *Klebsiella* predominantly and relatively low level of gammaproteobacteria than those in full term infants [46].

Breastfeeding have been reported to influence the infant microbiota. It was reported that microbiome involved the effects of DNA methylation through breast milk, which influences the gut microbiome community composition. Breastfeeding during this period is associated with greater *Bacteroides* and *Bifidobacterium*, which are folate producers, thereby affecting DNA methylation regulated by methyl-donor [47]. Also, breast milk oligosaccharides alter hut microbiome community that secrete short-chain fatty acid. Therefore, the strain of *Bifidobacterium* and *Lactobacillus* by breastfeeding could make intestinal contents more acidic with short-chain fatty acids, which modulate a defense mechanism against pathogens and epigenetic effects [48].

## Maternal Nutrients Influence on Microbiome and Epigenetic Modulation

As mentioned above, early environmental factors such as nutrition may have interacted with gut microbiome. Also, the microbial metabolites such as B vitamins, short chain fatty acids (SCFAs), polyphenols (ellagic acid, urolithin, equol, isothiocyanate), and omega3 polyunsaturated fatty acid (PUFA) are reported to influence epigenetic mechanisms [49,50]. Maternal gut microbe metabolites can change the host cellular levels of important epigenetic modifiers like histone acetyl transferases (HATs), histone deacetylases (HDACs), DNA methyltransferases (DNMTs), and DNA demethylases [51].

A number of the vitamins cannot be synthesized by the human body, therefore must be obtained by the diet. The gut microbiome is one of the important sources of B vitamins for the host [52]. Vitamins B2, B6, B9, and B12 are important cofactors for the enzymes in the folate cycle where the conversion from homocysteine to methionine is required for the availability of SAM. Therefore, these vitamins could affect histone methyl transferasess and DNMTs for histone and DNA methylation. The other gut microbial metabolites such as vitamins B3, B5, and SCFAs are sources of acetyl-CoA or NAD, which affect histone acetylation via sirtuin inhibition or HAT activation [51].

The microbial SCFAs from the fermentation of dietary fiber were shown to maintain the nervous and immune systems through epigenetic modification [17,53]. The SCFA acetate and butyrate are the most abundant in the intestinal tract and can be produced with acetyl-CoA which is universal acetyl group donor for histone acetylation. Maternal acetate suppressed asthma by Treg and Foxp3 through HDAC9 inhibition [54]. Besides, the SCFA butyrate induced global histone acetylation and activation in *FOXO3A* and *MT2* by inhibiting HDAC1 and HDAC2 [55]. The SCFA pentanoate produced by gut microbiota such as *Bacteroides, Bifidobacterium*, and *Lactobacillus* affects the use of the acetyl-CoA pool for histone acetylation [56] that inhibits HDAC1 and HDAC8 in CD4^+^ T cell, thereby reduce interleukin (IL)-17A production and enhance IL-10 production [56].

Polyphenols have antioxidant, anti-inflammatory, and immune-modulatory effects. Maternal supplementation of polyphenols improved the early development of the risk of intrauterine growth restriction [57]. Urolithins are microbial metabolites from ellagic acid (one of the polyphenols) that are reported to have a protective effect on chronic diseases such as metabolic syndrome [58] and decrease triglyceride accumulation in adipocytes [14]. Ellagic acid was reported to inhibit reduction of HDAC activity and urolithins prevented HAT activity [15]. Recent studies were shown that urolithin bacteria are *Bifidobacterium, pseudocatenulatum, Lactobacilli*, and *Coriobacteriaceae (Gordonibacter)* [59] and *Eggerthellaceae* family [60]. Equol produced by the intestinal bacterium *Lactococcus* reduced the risk of cardiovascular diseases including low-density lipoprotein cholesterol and arterial stiffness [61].

Omega-3 PUFAs are also obtained by the diet and have been interacted with gut microbiota. Maternal omega-3 PUFAs are a key role in the immune system of the infant through the epigenetic mechanism for DNA and histone methylation [11,62]. For the epigenetic mechanism, it was reported that maternal omega-3 PUFA induced changing of methylation level in differentially methylated regions [12] and the promoter methylation level of Interferon γ and IL13 [62]. Maternal omega-3 PUFAs regulated offspring obesity through recomposition of the gut microbiota, *Epsilonproteobacteria, Bacteroides, Akkermansia*, and *Clostridia* [63]. High- fat diet promotes intestinal epithelial HDAC3 level and SCFA butylate supplementation reduced HDAC3 activity [64]. Similarly, maternal high fat diet decreased fecal SCFA propionate and butylate level and increased *Firmicutes* to *Bacteroidetes* ratio, *Akkermansia*, and *Verrucomicrobia* in the offsprings related to blood pressure [65].

Maternal over-nutrition and undernutrition have shown to increase predisposition to obesity and epigenetic changes in offspring [2,66]. Such maternal malnutrition disrupted the stable microbial community, and less bacterial richness and diversity, which is linked to increased risk of inflammatory diseases and obesity [67-70]. The maternal high fat diet was associated with alterations in the gut microbiome profiles of offspring for *Coprococcus, Coriobacteriacae, Helicobacterioceae*, and *Allobaculum* [71], the ratio of *Bacteroidetes* to *Firmicutes* [72] in mice, *Campylobacter* in Macaca fuscata [13] and *Bacteroides* in humans [73]. For the epigenetic mechanism, it was reported that the high-fat diet affected HNF4ɑ binding sites at acetylated histone H3K27 in colon epithelium [10] and decreased *Bifidobacteriaceae* [74]. Similarly, over-weighted woman during pregnancy had higher *Bacteroids* and lower *Phascolarctobacterium* than a normal-weighted woman during pregnancy [75]. Under-nutrition group in school-age children had a higher level of the *Firmicutes* and *Lachnospiraceae* [76]. Besides, maternal supplementation of probiotics with *Lactobacillus rhamnousus* or *Bifidobacterium lactis* induced interferon-gamma production on cord blood compared to controls [77].

## Conclusion

The interaction between the developing gut microbiome and epigenetic processes is an important mechanism of developmental reprogramming of immune disorder, obesity, and metabolic disorders. There are mounting evidence supporting that gut microbiota may modify the pattern of DNA methylation and histone modification. Recent studies have shown that the gut microbiome produces plenty of metabolites come from maternal nutrients that have the potential to modulate DNA methylation and histone modification. Maternal nutrients may have a crucial role in early development and long term health consequences. This review explored the impact of maternal nutrients on epigenetic mechanisms that regulate the early-life microbiome profile. The importance of individual nutrients induced by epigenetic modulation will help achieve optimal gut microbiota and strategy for improving health consequences.

## Figures and Tables

**Table 1.** Epigenetic modifications and their functions with nutrients

Target	Residue	Modification	Major function	Related nutrients	Related microbiome	Reference
DNA	CpG	me	Transcriptional repression	Folate, choline, omega3-PUFA, polyphenols, proteins, high-fat diet	Bifidobacterium	[7,8]
					Lactobacterium	
					Bacteroids	
	K4	me1, me2, me3	Transcriptional activation, poised to transcriptional activation	Low-fat diet, riboflavin, polyphenols, cobalamin	Firmicutes	[9,10]
					Lachnospiraceae	
	K27	me1	Transcriptional activation	Omega3-PUFA	Firmicutes	[11-13]
				high-fat diet	Bacteroidestes	
				Nicotinamide	Akkermansia	
		me2	Transcriptional activation, enhancer silencing		Verrucomicrobia	
		me3	Transcriptional repression		Bifidobacteriaceae	
	K9, K4, K14, K36	ac	Transcriptional activation	Omega3-PUFA, selenium, SCFAs, ellagic acid, high-fat diet	Bifidobacterium	[9,14,15]
					Anaerostipes	
					Eubacterium	
Histone H4	K16	ac	Transcriptional activation and repression for DNA repair	Curcumin	Clostridium	[16]
					Firmicutes	
					Bacteriodetes	
	K4, K8, K12	ac	Transcriptional activation	SCFAs, folate, biotin	Bifidobacterium	[17]
					Lactobacillus	
					Bacteroides	

me, methylation (1, mono; 2, bi; 3, tri); PUFA, polyunsaturated fatty acid; ac, acetylation; SCFA, short chain fatty acid.
